# Morphology, Chemistry, and Phylogeny Reveal Two New Species of the Genus *Lecidella* (Ascomycota, Lecanoraceae) from Anhui Province, China

**DOI:** 10.3390/jof12060453

**Published:** 2026-06-22

**Authors:** Yi-Shan Feng, Xin-Yu Wang, Yan-Yun Zhang

**Affiliations:** 1 Key Laboratory of Biodiversity Conservation and Ecological Security in the Yangtze River Basin of Anhui Province, College of Life Sciences, Anhui Normal University, Wuhu 241000, China; 2421011726@ahnu.edu.cn; 2 State Key Laboratory of Phytochemistry and Natural Medicines, Kunming Institute of Botany, Chinese Academy of Sciences, Kunming 650201, China; wangxinyu@mail.kib.ac.cn; 3 Yunnan Key Laboratory for Fungal Diversity and Green Development, Kunming Institute of Botany, Chinese Academy of Sciences, Kunming 650201, China

**Keywords:** eastern China, lecideine apothecia, lichen, taxonomy

## Abstract

In this study, two new species, *Lecidella biformis* Y. S. Feng & Y. Y. Zhang and *L. versicolor* Y. S. Feng & Y. Y. Zhang, are described from Anhui Province, China, based on morphological, chemical, and molecular evidence. Phylogenetic analyses show that these two new species, together with *L. albida* Hafellner, form a well-supported clade characterized by small apothecia (less than 0.5 mm in diameter), a hyaline, yellowish-brown or nut-brown hypothecium, and the presence of atranorin. *Lecidella biformis* is distinguished by dimorphic apothecia, ranging from brown with a paler margin to entirely brown, a blackish-brown or nut-brown epithecium, a yellowish-brown or nut-brown hypothecium, and the presence of atranorin and methyl 2′-O-methylmicrophyllinate. *Lecidella versicolor* is characterized by apothecia that are yellow to reddish-brown when young and nut-brown at maturity, an orange-brown epithecium, a hyaline to yellowish-brown hypothecium, anastomosed paraphyses, and the presence of atranorin only. Detailed descriptions, discussions, and illustrations are provided for the new species, along with a key to all known *Lecidella* species from China.

## 1. Introduction

*Lecidella* was established by Körber [1], who separated it from *Lecidea* Ach. based on its lighter-colored, never carbonaceous hypothecium. Nevertheless, only a few lichenologists initially accepted this concept [2,3] and, for a long time, these species were largely considered part of the genus *Lecidea* Ach. [4]. Later, Leuckert and Hertel [5] re-elevated *Lecidella* to generic rank, based on the presence of chlorinated norlichexanthones in many species. Currently, *Lecidella* is classified within the family Lecanoraceae, not only because of similarities in ascus type [6,7] but also based on strong support from molecular data [8,9]. The genus is characterized by dark-colored lecideine apothecia; a persistent proper excipulum; clavate, large amyloid, eight-spored asci of the *Lecidella*-type; simple, hyaline, non-halonate ascospores; curved filiform conidia; and the presence of xanthones in the majority of species [8,10,11]. To date, the genus *Lecidella* includes approximately 80 accepted species worldwide [12], of which 14 have been recorded in China [13,14]. The genus is widely distributed and occurs on various substrates [8,11,15,16].

Anhui Province is located in eastern China, situated in the transitional zone between the Huai River basin and the lower Yangtze River basin [17]. Previously, only two *Lecidella* species, *L. elaeochroma* (Ach.) M. Choisy and *L. euphorea* (Flörke) Kremp., had been recorded in the province [18]. Over the past five years, we have conducted more than ten field surveys targeting *Lecidella* and collected several specimens with small apothecia that could not be assigned to any known species, along with two newly recorded taxa from Anhui Province, *Lecidella mandshurica* S.Y. Kondr., Lőkös & Hur and *L.* aff. *euphorea*, found during this study. Based on integrated morphological, chemical, and molecular phylogenetic evidence, we here describe these specimens as two new species of *Lecidella* from Anhui Province, China.

## 2. Materials and Methods

### 2.1. Morphology and Chemistry

We collected six specimens of *Lecidella biformis* and one specimen of *Lecidella versicolor* from the southeastern part of the Dabie Mountains in Anhui Province, China. These specimens are preserved in the Botany Herbarium of Anhui Normal University (ANUB). A key to the *Lecidella* species from China is provided here.

External morphological features were observed, measured, and described using an OLYMPUS SZ61TR (Hachioji, Japan) stereomicroscope. Key characteristics were photographed with a Mingmei MS60-2 (Guangzhou Mingmei Optoelectric Technology Co., Ltd., Guangzhou, China) camera. Thin sections of apothecia were manually prepared using a razor blade, mounted in water, and examined under an OLYMPUS BX43 (Hachioji, Japan) compound microscope to observe anatomical structures. Photomicrographs were taken using a Mingmei MDX10 (Guangzhou, China) camera. The colors of the thallus surface, apothecia, and vertical sections of apothecia were compared and identified using the RAL color chart (Bonn, Germany). Spore measurement data are presented as follows: [minimum] − ($\bar{x}$ − SD) − $\bar{x}$ − ($\bar{x}$ + SD) − [maximum], where $\bar{x}$ is the arithmetic mean and SD is the standard deviation (values rounded to the nearest 0.5 µm), followed by the number of measurements (*n*) [19]. A 10% Lugol’s iodine solution (I) was used to examine the apical structure of asci. Crystals in apothecia were observed under polarized light (POL); their solubility was tested in 20% nitric acid (HNO_3_ = “N”) and 10% potassium hydroxide (KOH = “K”), where N-sol/K-sol indicates that crystals dissolved and N-insol/K-insol indicates that they did not dissolve.

Spot tests were conducted using K, C (10% calcium hypochlorite), and KC (K followed by C). To assess compositional differences between the thallus and apothecia, approximately 20 mg of thallus and 4–5 apothecia were separately sampled from each specimen and analyzed using thin-layer chromatography (TLC) with solvent systems A and C, according to the methods of [5,20].

### 2.2. DNA Extraction, PCR and Sequencing

Apothecia from dry specimens were taken to extract genomic DNA using the Chelex^®^ 100 Resin (Bio-Rad, Hercules, CA, USA) method [21] following the manufacturer’s instructions. For PCR amplification of the nrITS region, primers ITS1F and ITS4a were employed [22,23], while for the nrLSU region, primers LR0R and LR5 were used [24,25]. The PCR reaction mix (total volume 25 µL) contained the following: 9.5 μL dd H_2_O, 12.5 μL 2 × Trio Taq Master Mix (Monad, Anhui, China), 1 μL of each primer, and 1 μL of DNA. The PCR amplification conditions for both nrITS and nrLSU were as follows: initial denaturation at 94 °C for 5 min; 35 cycles of denaturation at 94 °C for 15 s, annealing at 53 °C for 15 s, and extension at 72 °C for 1 min; final extension at 72 °C for 10 min; hold at 4 °C. The PCR products were sent to General Biosystems (Chuzhou, China) for sequencing.

### 2.3. Phylogenetic Analyses

Using the NCBI online BLAST tool (https://blast.ncbi.nlm.nih.gov/Blast.cgi (accessed on 7 December 2024)), we analyzed the raw sequences to confirm that the DNA belonged to lichenized fungi, made a preliminary assessment of their taxonomic status, and downloaded additional *Lecidella* sequences from GenBank for phylogenetic tree construction (Table 1). Following the phylogenetic framework of *Lecidella* [8], *Protoparmelia badia* (Hoffm.) Hafellner and *P. picea* (Dicks.) Hafellner were selected as the outgroup for our analysis.

Geneious 2025.0.2 was used to assemble and edit the raw sequences and to generate a single matrix for nrITS and nrLSU. Each sequence was aligned using the online MAFFT service (https://mafft.cbrc.jp/alignment/server/index.html (accessed on 30 March 2026)). Prior to concatenating the single-gene datasets, we tested for potential incongruence in PhyloSuite v1.2.3 [26] using 1000 ultrafast bootstrap replicates. The nrITS and nrLSU genes were then combined using the concatenate sequence function after confirming that all statistically supported clades (bootstrap ≥ 95%) were consistent across the individual gene trees.

Maximum likelihood (ML) and Bayesian inference (BI) analyses based on the two loci were performed using PhyloSuite v1.2.3 [26]. ModelFinder [27] was used to estimate the best partitioning schemes and nucleotide substitution models. The IQ-TREE best-fit models were TIM2 + F + G4 for ITS1 and ITS2, TNe + R2 for 5.8S, and nrLSU. Statistical support values were obtained through 1000 standard non-parametric bootstrap replicates, with default settings used for other parameters. The MrBayes best-fit models were GTR + F + G4 for ITS1 and ITS2, SYM + I + G4 for 5.8S, and nrLSU. Four chains (three heated and one cold) were used and were run for 2 million generations. Samples were taken every 1000th generation, and the burn-in was set to 0.25 (i.e., the first 25% of samples were discarded). Bayesian convergence was considered achieved when the average standard deviation of split frequencies (ASDSF) was below 0.01. All other parameters were set to default values. Support values were annotated on branches when the Shimodaira–Hasegawa-like approximate likelihood ratio test (SH-aLRT) was ≥80%, maximum likelihood bootstrap values (MLBPs) were ≥70% and Bayesian posterior probabilities (BPPs) were ≥0.90. The phylogenetic tree was visualized using FigTree v.1.4.4 and subsequently refined and edited in Adobe Illustrator 2020 SP.

**Table 1:** Sequences used in this study. Newly obtained sequences are in bold. “na” indicates that no sequence is available and “—” means that the sequences were submitted directly to NCBI.

Species Name	Country	Voucher Specimens	GenBank Accession Number		Reference
			nrITS	nrLSU	
*Lecidella achristotera*	Austria	Vondrák 26094 (PRA)	OQ717476	na	[28]
*L. achristotera*	Czech Republic	Vondrák 25559 (PRA)	OQ717918	na	[28]
*L.* aff. *euphorea*	China: Anhui	ZYY 24-1733 (ANUB)	**PZ322664**	**PZ319260**	This paper
*L.* aff. *euphorea*	China: Yunnan	ZX YN 0186-2	KT453755	KT453782	[9]
*L.* aff. *euphorea*	China: Shaanxi	ZX 20114605	KT453756	KT453781	[9]
*L.* aff. *flavosorediata*	Russia	J. Malicek 10904 (hb. JM)	MK778614	na	[29]
*L. albida*	Switzerland	LIFU 055-16	KX132964	na	[30]
*L. albida*	Czech Republic: Krivoklatsko	ZP 30064 (PRA)	OL457926	na	[31]
*L. albida*	Canada: Alberta, Edmonton	D. Haughland 2020-28 (hb. Haughland)	ON116032	na	[32]
*L. albida*	Czech Republic	Palice33684 (PRA)	OQ717477	na	[28]
*L. asema* var. *elaeochromoides*	China: Shaanxi	ZX 20114966-2	KT453746	KT453948	[9]
*L. asema* var. *elaeochromoides*	China: Xinjiang	ZX 20141142	KT453750	KT453790	[9]
*L. ayazii*	Antarctica: James Ross Island	JR 0.062 (ERCH)	OQ534850	na	[11]
*L. ayazii*	Antarctica: James Ross Island	JR 0.323 (ERCH)	OQ534851	na	[11]
*L. biformis*	China: Anhui	ZYY 24-1546 (ANUB)	**PZ322658**	**PZ319255**	This paper
*L. biformis*	China: Anhui	ZYY 24-1551 (ANUB)	**PZ322659**	**PZ319256**	This paper
*L. biformis*	China: Anhui	ZYY 24-1692 (ANUB)	**PZ322660**	**PZ319257**	This paper
*L. biformis*	China: Anhui	ZYY 24-1699 (ANUB)	**PZ322661**	**PZ319258**	This paper
*L. biformis*	China: Anhui	ZYY 24-1746 (ANUB)	**PZ322662**	**PZ319259**	This paper
*L. biformis*	China: Anhui	ZYY 24-1752 (ANUB)	**PZ322663**	na	This paper
*L. carpathica*	China: Inner Mongolia	ZX 20141477	KT453739	KT453783	[9]
*L. carpathica*	China: Xinjiang	ZX 20140367-2	KT453741	KT453784	[9]
*L.* cf. *wulfenii*	USA: Alaska	Tønsberg 43815 (BG)	MN906295	na	[33]
*L. drakensis*	Chile: Region de Magallanes y de la Antartica Chilena	UR 00120 (SZU)	MK620158	na	[11]
*L. drakensis*	Antarctica: James Ross Island	JR 0.082 (ERCH)	OQ534854	na	[11]
*L. effugiens*	China: Jilin	ZX 20141148-2	KT453747	KT453786	[9]
*L. effugiens*	China: Jilin	ZX 20141269-2	KT453748	KT453785	[9]
*L. elaeochroma*	Germany: Hessen	FR-0261123	PQ099899	PQ100515	[34]
*L. elaeochroma*	China: Xinjiang	ZX XL0395-2	KT453749	KT453789	[9]
*L. enteroleucella*	China: Yunnan	ZX YN0201	KT453757	KT453792	[9]
*L. euphorea*	Spain	ABG 25	OR105762	na	[35]
*L. euphorea*	China: Xinjiang	ZX 20140638	KT453742	KT453798	[9]
*L. euphorea*	China: Xinjiang	ZX XL 0351-2	KT453745	KT453797	[9]
*L. flavosorediata*	Switzerland	LIFU 056-16	KX132965	na	[30]
*L. flavosorediata*	Spain	ABG 47	OR105778	na	[35]
*L. fuliginea*	Brazil	A. Aptroot 52200 (L.A. dos Santos)	ON178677	na	[36]
*L. greenii*	Antarctica: Victoria Land	R.Türk 33612 (SZU)	JN873884	na	[37]
*L. greenii*	Antarctica: Ross Dependency	T 44636	MK208776	na	[38]
*L. iqbalii*	Pakistan	Afshan & Niazi 37007 (LAH)	OL843365	na	[39]
*L. laureri*	Finland	59388	OR075190	na	[40]
*L. leprothalla*	Switzerland	LIFU 057-16	KX132966	na	[30]
*L. mandshurica*	China	ZYY 24-1079 (ANUB)	**PZ322665**	na	This paper
*L. mandshurica*	China	ZYY 24-1318 (ANUB)	**PZ322666**	na	This paper
*L. mandshurica*	China	ZYY 24-1583 (ANUB)	**PZ322667**	na	This paper
*L. mandshurica*	China	ZYY 24-1596 (ANUB)	**PZ322668**	na	This paper
*L. mandshurica*	China	ZYY 21-115 (ANUB)	**PZ322669**	na	This paper
*L. mandshurica*	China: Jilin	ZX 20141284	KT453751	KT453780	[9]
*L. mandshurica*	South Korea: Gangwon-do	Kondratyuk & L. Lőkös 034385 (KoLRI)	MK672836	na	[41]
*L. meiococca*	Sweden	Ekman 3101 (BG)	AF517929	na	[42]
*L. patavina*	China: Xinjiang	ZX XL 0345	KT453761	KT453801	[9]
*L. patavina*	China: Xinjiang	ZX 20140501-2	KT453767	KT453799	[9]
*L. scabra*	China	NALH-XT 0609 (XJU)	PV688700	na	—
*L. scabra*	China	NALH-XT 0612 (XJU)	PV688702	na	—
*L. siplei*	Antarctica: Victoria Land	R. Türk 32987 (SZU)	JN873895	na	[37]
*L.* sp	Portugal	Sipman 62831	MN586977	na	[43]
*L.* sp	Portugal	Sipman 62998	MN586978	na	[43]
*L.* sp	Portugal	Sipman 62989	MN586979	na	[43]
*L.* sp	Czech Republic	Malicek 14713 (hb. JM)	OP730574	na	[44]
*L.* sp	Czech Republic	Palice 32481 (PRA)	OQ717478	na	[28]
*L.* sp. 1	Argentina: Provincia de Chubhut	UR 00211	MK620219	na	[45]
*L.* sp. 1	Argentina: Provincia de Chubhut	UR 00213	MK620221	na	[45]
*L.* sp. 1	Antarctica: Maritime Antarctica	UR 00970	PV788476	na	[16]
*L.* sp. 1	Antarctica: Maritime Antarctica	UR0 0985	PV788477	na	[16]
*L.* sp. 2	Antarctica: Ross Dependency	GS1_64 (MAF)	MK208746	na	[45]
*L.* sp. 2	Antarctica: Ross Dependency	HS7_59 (MAF)	MK208747	na	[38]
*L.* sp. 3	Antarctica: Maritime Antarctica	UR 00860	PV788446	na	[16]
*L.* sp. 3	Antarctica: Maritime Antarctica	UR 00879	PV788447	na	[16]
*L.* sp. 4	Antarctica: Maritime Antarctica	UR 00896	PV788479	na	[16]
*L.* sp. 4	Antarctica: Maritime Antarctica	UR 00908	PV788480	na	[16]
*L. stigmatea*	China: Xinjiang	ZX 20141254	KT453758	KT453808	[9]
*L. stigmatea*	China: Xinjiang	ZX 20140086-2	KT453764	KT453804	[9]
*L. tumidula*	China: Xinjiang	ZX XL 0009	KT453736	KT453810	[9]
*L. tumidula*	China	NALH-XT0024b (XJU)	PV688674	na	—
*L. versicolor*	China: Anhui	ZYY 24-1504 (ANUB)	**PZ322657**	**PZ319254**	This paper
*L. wulfenii*	Austria: Salzburg	R. Türk 39666 (SZU)	JN873903	na	[37]
*L. yunnanensis*	China: Yunnan	A. H. Ekanayaka 17-1910-a (MFLU)	MK075945	MK075949	[13]
*L. yunnanensis*	China: Yunnan	A. H. Ekanayaka 17-1910-b (MFLU)	MK075946	MK075950	[13]
*Protoparmeli badia*	USA	Fryday 8575	KY066254	KY066280	[46]
*P. picea*	Norway: Sor-Trondelag	Haugan 9612 (O)	KF562194	KF562186	[47]

## 3. Results and Discussion

**Figure 1:**
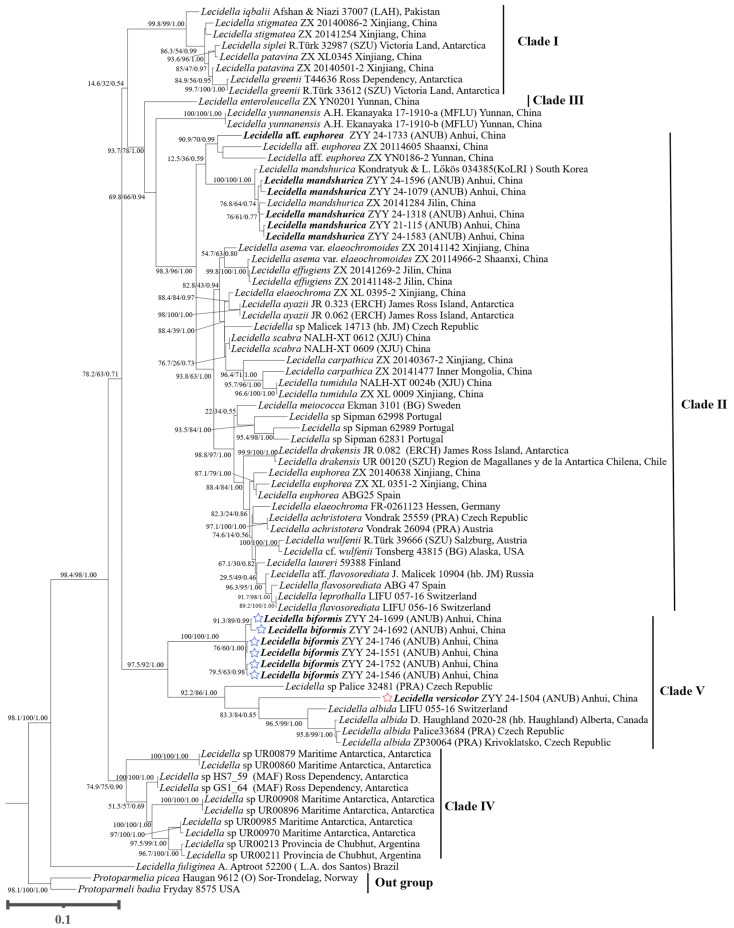
Maximum likelihood (ML) phylogenetic tree based on the concatenated nrITS and nrLSU dataset. Support values (SH-aLRT ≥ 80%/ML bootstrap values ≥ 70%/Bayesian posterior probabilities ≥ 0.90) are displayed along the branches. Newly generated sequences are indicated in bold, and the two new taxa are marked with pentagrams.

The concatenated nrITS-nrLSU alignment comprised 108 sequences, including 89 obtained from GenBank and 19 newly generated in this study (Table 1). This dataset encompasses the majority of available sequences of *Lecidella* species. Both methods produced congruent tree topologies; the ML tree is presented in the main text (Figure 1).

The two-locus phylogenetic tree shows that species of the genus *Lecidella* fall into five main clades and two distinct species lineages, although the relationships between these clades are weakly supported (Figure 1). Clades I–IV are consistent with those from previous studies [8,45], and we identify an additional new lineage, Clade V (97.5/92/1.00), here. Species of Clade I are characterized by a hyaline hypothecium and the absence of xanthones. Clade II consists of several subclades and species lineages, with most species having a yellowish-brown, reddish-brown, or brown hypothecium and containing xanthones. Clade III comprises a single species, *L. enteroleucella* (Nyl.) Hertel, which is distinguished by a colorless hypothecium and produces the chlorinated xanthones, thuringione and arthothelin [8]. Clade IV mainly includes specimens from southern South America (sSA) and continental Antarctica; however, no detailed morphological or chemical characteristics have been provided for these undescribed species [16,45].

Species in Clade V, which is newly proposed here, are characterized by small apothecia (less than 0.5 mm in diameter), a hyaline, yellowish-brown, or nut-brown hypothecium, and the presence of atranorin. Both new species belong to this clade. *Lecidella biformis* (100/100/1.00) represents the basal lineage of Clade V, and is distinguished by its dimorphic apothecia, yellowish-brown or nut-brown hypothecium, as well as the presence of atranorin and methyl 2′-O-methylmicrophyllinate. *Lecidella versicolor* forms a single-sample lineage that is sister to *L. albida* and clusters with an unidentified sample from the Czech Republic. However, *L. albida* differs in having a sorediate thallus, a bluish-grey to blackish-brown epithecium, and the presence of atranorin, capistratone, and thiophanic acid [48]. In our phylogenetic analysis, another species with small apothecia, *L. yunnanensis* Ekanayaka & K.D. Hyde, is sister to Clade II and is clearly distinct from other known *Lecidella* species. It is characterized by its black, shiny apothecia; blackish-brown hypothecium; and 1-septate ascospores, which are hyaline when immature and greenish-brown at maturity [13].

Six additional *Lecidella* species also possess small apothecia (less than 0.5 mm in diameter), but their sequences are not yet available: *L. aptrootii* Knoph & Garnitz, *L. commutata* Knoph & Leuckert, *L. nashiana* Knoph & Leuckert, *L. oceanica* Lu L. Zhang & Xin Y. Wang, *L. subviridis* Tønsberg, and *L. varangrica* Haugan & Tønsberg [49,50,51,52,53]. Among these, *L. aptrootii*, *L. oceanica*, *L. subviridis*, and *L. varangrica* share the same combination of characters—a hyaline or yellowish-brown hypothecium and the presence of atranorin [49,51,52,53]—as the species of Clade V; therefore, we hypothesize that they will also belong to this clade. The hypothecium of *L. commutata* and *L. nashiana* is also hyaline or weakly yellowish-brown, but these species lack atranorin [49,50], suggesting that they may represent a lineage distinct from Clade V. The phylogenetic placement of these species requires clarification in future studies. Nevertheless, our new species can be readily distinguished from aforementioned species by thallus and apothecial characters, as well as by differences in secondary chemistry (Table 2).

**Table 2:** Comparison of diagnostic characters among *Lecidella* species possessing small apothecia (less than 0.5 mm in diameter).

Species	External Morphology	Epithecium	Hypothecium	Chemistry	Substrate	Reference
*Lecidella albida* Hafellner	Thallus thin, sometimes sorediate; apothecia 0.2–0.5 mm; disc nut-brown to black.	Bluish-grey to blackish-brown	Hyaline to yellowish-brown	Atranorin, capistratone, thiophanic, arthothelin (±trace)	On bark	[48]
*L. aptrootii* Knoph & Garnitz	Thallus coherent to rimose; apothecia up to 0.2–0.25 (–0.33) mm, disc black.	Pale green to blackish-brown	Hyaline	Atranorin, aotearone, capistratone	On branches and twigs	[49]
*L. biformis* Y. S. Feng & Y. Y. Zhang	Thallus thin, continuous to rimose to nearly granulose; apothecia 0.05–0.25 mm; disc dimorphic.	Blackish-brown or nut-brown	Yellowish-brown or nut-brown	Atranorin, methyl 2′-O-methylmicrophyllinate	On bark	This paper
*L. commutata* Knoph & Leuckert	Thallus coherent to areolate; apothecia 0.2–0.4 (–0.5) mm; disc black.	Hyaline to pale yellowish olive-grey	Hyaline	Two chemotypes: (a) containing vicanicin, vicanicinmonomethylether(trace); (b) vivanicin, 2,5,7-trichloro-3-O-methylnor lichexanthone (trace)	On bark	[49]
*L. nashiana* Knoph & Leuckert	Thallus continuous to rimose to nearly granulose; apothecia 0.2–0.4 mm; disc black.	Green, blackish-green, bluish-green, blackish-brown	Weakly yellowish-brown	2,7-dichloro-6-O-methylnorlichexanthone, 2′-O-methylperlatolic acid	On bark	[50]
*L. oceanica* Lu L. Zhang & Xin Y. Wang	Thallus rimose to areolate; apothecia up to 0.3 (–0.4) mm; disc brownish-black to black.	Olive, olive-brown to brown	Hyaline	Atranorin(±), capistratone, isoarthothelin and thiophanic acid.	On large siliceous boulders	[51]
*L. subviridis* Tønsberg	Thallus areolate to continuous, sorediate, sometimes leprose; apothecia 0.1–0.4 (–0.5) mm; disc black.	Brown	Hyaline	Atranorin, thiophanic acid, arthothelin, expallens unknown	On trunk, branches and twigs	[52]
*L. varangrica* Haugan & Tønsberg	Thallus episubstratal, areolate; apothecia 0.2–0.5 mm; disc black.	Bluish-grey to blackish-brown	Brown to yellowish-brown	Aotearone, capistratone, thiophanic acid, isoarthothelin, and atranorin (trace)	On rock	[53]
*L. versicolor* Y. S. Feng & Y. Y. Zhang	Thallus thin, continuous to rimose; apothecia 0.1–0.25 mm; disc yellow to reddish-brown when young, nut-brown at maturity.	Orange-brown	Hyaline to yellowish-brown	Atranorin	On bark	This paper
*L. yunnanensis* Ekanayaka & K.D. Hyde	Thallus continuous to nearly granulose; apothecia 0.3–0.5 mm; disc black.	Blackish-brown	Blackish-brown	Not provided	On bark	[13]

### 3.1. Taxonomy

***Lecidella biformis* Y. S. Feng & Y. Y. Zhang, sp. nov**.

Fungal name: FN573735

Figure 2A–H

**Etymology.** The epithet refers to its dimorphic apothecia.

**Type.** CHINA • Anhui Prov, Lu’an City, Jinzhai County, Madiedang Village, 31°32′00″ N, 115°26′31″ E, 381 m alt., on *Liquidambar formosana* bark, 4 November 2024, Yanyun Zhang, Yujiao Yin and Yishan Feng 24-1752 (ANUB2412)—***holotype***.

**Diagnosis.** The species is characterized by its small, dimorphic apothecia—even within a single specimen—with either a brown disc and paler margin or a purely brown disc and margin, a blackish-brown or nut-brown epithecium, a yellowish-brown or nut-brown hypothecium, and the presence of atranorin and methyl 2′-O-methylmicrophyllinate.

**Description.** Thallus crustose, thin, continuous to rimose to nearly granulose; upper surface pale green or patina-green; prothallus absent; apothecia lecideine, dispersed to aggregated, orbicular, adnate, 0.05–0.25 mm in diameter, disc plane, margin persistent, entire, dimorphic even in a single specimen: disc brown with paler margin or disc and margin purely brown. Exciple orange-brown or nut-brown, 15–32.5 (−37.5) µm thick, crystals present (pol+, K-sol, N-insol) or absent; parathecium indistinct; epithecium blackish-brown or nut-brown, (2.5–) 5.0–10.0 µm thick, crystals present (pol+, K-sol, N-insol) or not; epihymenium pale brown to nut-brown, sometimes suffused with pigment of epithecium in the upper part, 12.5–25 µm high; hymenium pale brown to nut-brown, 22.5–45.0 (−50.0) µm high; hypothecium yellowish-brown or nut-brown, 7.5–15.0 (−17.5) µm high; paraphyses simple to branched, not anastomosing, 1.5–2 µm thick; asci *Lecidella*-type, clavate, usually immature, 25.0–50.0 × 5.0–15.0 µm, 8-spored; ascospores simple, hyaline, ellipsoid to widely ellipsoid, [3.5]–(5.8)–7.5–(9.2)–[11.0] × [2.5]–(3.5)–5.0–(6.5)–[7.0] µm (*n* = 50). Pycnidia unknown.

**Chemistry.** Thallus K+ yellow, C-, KC+ yellow. Both thallus and apothecia contain atranorin and methyl 2′-O-methylmicrophyllinate.

**Distribution.** The new species has been found on the *Liquidambar formosana* bark or dead wood at altitudes between 362 and 807 m in Anhui Province, China.

**Notes.** The new species shares small apothecia and pigmented hypothecium with *Lecidella nashiana*, *L. varangrica,* and *L. yunnanensis*. However, *L. nashiana* is distinguished by its black disc; its green, blackish-green, or bluish-green epithecium; and the production of 2,7-dichloro-6-O-methylnorlichexanthone and 2′-O-methylperlatolic acids [50]. *Lecidella varangrica* differs by its sorediate thallus, black disc, and saxicolous habitat [53], while *L. yunnanensis* is distinguished by its black, shiny apothecia and its 1-septate ascospores, which are hyaline when immature and greenish-brown at maturity [13].

**Additional specimens examined:** CHINA • Anhui Prov, Lu’an City, Jinzhai County, Baojiawo Forest farm, Longjing River, 31°13′27″ N, 115°51′26″ E, 629 m alt., on *Liquidambar formosana* bark, 3 November 2024, Yanyun Zhang, Yujiao Yin and Yishan Feng 24-1699 (ANUB2359); Dahuanglishuling, 31°10′10″ N, 115°50′42″ E, 798–807 m alt., on *Liquidambar formosana* bark, 7 August 2024, Yanyun Zhang and Xiaoying Wu 24-1546 (ANUB1932), 24-1551 (ANUB1937); Dahuanglishuling, 31°10′09″ N, 115°50′37″ E, 795 m alt., on dead wood, 2 November 2024, Yanyun Zhang, Yujiao Yin and Yishan Feng 24-1692 (ANUB2352); Madiedang Village, 31°32′00″ N, 115°26′31″ E, 362 m alt., on *Liquidambar formosana* bark, 4 November 2024, Yanyun Zhang, Yujiao Yin and Yishan Feng 24-1746 (ANUB2406).

***Lecidella versicolor* Y. S. Feng & Y. Y. Zhang, sp. nov**.

Fungal name: FN573734

Figure 3A–I

**Etymology.** The specific epithet refers to the variable color of the apothecial disc, even within the same specimen.

**Type.** CHINA • Anhui Prov, Lu’an City, Jinzhai County, Xiaonanpeng, 31°16′02″ N, 115°38′52″ E, 496 m alt., on *Liquidambar formosana* bark, 6 August 2024, Yanyun Zhang and Xiaoying Wu 24-1504 (ANUB1890)—***holotype***.

**Diagnosis.** The new species is distinguished from other *Lecidella* species by its small apothecia, an orange-brown epithecium, a hyaline to yellowish-brown hypothecium, anastomosing paraphyses, and the presence of atranorin as the sole compound.

**Description.** Thallus crustose, thin, continuous to rimose; upper surface pale green, becoming white-gray in the herbarium; prothallus present, black-blue; apothecia lecideine, dispersed to in groups of 2–3, orbicular, adnate, 0.1–0.25 mm in diameter; disc plane, yellow to reddish-brown with indistinct to thin and same level margin when young, nut-brown with thick, entire and protruding margin at maturity. Exciple orange-brown, 12.5–25 µm thick, crystals present (pol+, K-sol, N-insol) or absent; parathecium indistinct; epithecium orange-brown, 5.0–12.5 µm thick, crystals present (pol+, K-sol, N-insol); epihymenium hyaline to weakly yellowish-brown, sometimes suffused with pigment of epithecium in the upper part, 12.5–25 µm high; hymenium hyaline to weakly yellowish-brown, 30.0–50.0 µm high; hypothecium hyaline to yellowish-brown, 10.0–25.0 (−30.0) µm high; paraphyses simple to rarely branched, anastomosing, ca. 1 µm thick; asci *Lecidella*-type, clavate, usually immature, 17.5–55.0 × 3.8–15.0 µm, 8-spored; ascospores simple, hyaline, broadly ellipsoid or ovoid, [7.0]–(8.1)–9.3–(10.5)–[12.0] × [3.0]–(4.6)–5.3–(6.0)–[6.5] µm (*n* = 34). Pycnidia unknown.

**Chemistry.** Thallus K+ yellow, C-, KC-. Both thallus and apothecia contain atranorin.

**Distribution.** This species occurs on the *Liquidambar formosana* bark and is known from Anhui Province, in the south-eastern part of the Dabie Mountains at 496 m altitude.

**Notes.*** Lecidella albida* is closely related to the new species and shares the characters of small apothecia (0.2–0.5 mm in diameter) and a hyaline to yellowish-brown hypothecium. However, *L. albida* differs in having a sorediate thallus, nut-brown to black apothecia, a bluish-grey to blackish-brown epithecium and containing capistratone and thiophanic acid as accessory compound alongside atranorin [48].

*Lecidella aptrootii*, *L. commutata*, *L. oceanica* and *L. subviridis* also have small apothecia and a hyaline hypothecium, but their apothecia are black, while *L. aptrootii* has a green or blackish-brown epithecium and contains the secondary metabolites atranorin, aotearone and capistratone [49]. *Lecidella commutata* is characterized by an areolate thallus, a colorless to pale yellowish olive-grey epithecium, and the presence of vicanicin [49]. *Lecidella oceanica* has a rimose or areolate thallus, an olive, olive-brown to brown epithecium, and is sometimes saxicolous [51]. *Lecidella subviridis* has a sorediate thallus, a brown epithecium, and contains thiophanic acid and expallens unknowns, as accessory compounds alongside atranorin [52].

*Lecidella destituta* Kantvilas & Elix and *Lecidella iqbalii* Fayyaz, Afshan, Niazi & Khalid often contain only atranorin; however, *L. destituta* differs by possessing a typically areolate, deeply cracked thallus, apothecia 0.2–0.9 mm in diameter and a pale yellow-brown to yellow-orange hypothecium [10]. *L. iqbalii* can be distinguished by its areolate thallus, its black apothecia 0.1–1.2 mm in diameter and its blackish-brown epithecium [39].

### 3.2. Key to the Species of Lecidella in China

1 On bryophytes                                                           *Lecidella wulfenii*

- On bark, wood, or rocks                                                               2

2 Apothecial discs black with shiny appearance; ascospores hyaline when immature, greenish-brown, 1-septate at maturity             *L. yunnanensis*

- Apothecia discs matt; ascospores hyaline, simple                                                    3

3 Apothecia discs plane, margin persistent, entire, dimorphic even in a single specimen: disc brown with paler margin or disc and margin purely brown *L. biformis*

- Apothecia not dimorphic                                                             4

4 Apothecial discs yellow to reddish-brown when young and nut-brown at maturity; both thallus and apothecia contain atranorin only        *L. versicolor*

- Apothecial discs brown, brownish-black to black, or black; containing other compounds                                   5

5 Hypothecium and parathecium hyaline or weakly yellowish-brown; on rocks                                       6

- Hypothecium and parathecium yellowish-brown, reddish-brown or brown; on bark or wood, or on rocks                             10

6 Zeorin absent                                                                    7

- Zeorin present                                                                   9

7 Apothecial discs pale gray to bluish-gray pruinose; thallus with usnic acid, ± psoromic acid, and terpenoids as accessory compounds together with atranorin 

                                                                       *L. bullata*

- Apothecial discs epruinose; usnic acid and terpenoids absent                                               8

8 Apothecia discs flat, margin distinct; prothallus absent or black; containing capistratone, isoarthothelin and thiophanic acid as accessory compound alongside atranorin                                                                    *L. oceanica*

- Apothecial discs often become convex with an obliterated margin; prothallus gray or blackish-gray to black; thallus with arthothelin and thuringione as accessory compounds together with atranorin                                                      *L. enteroleucella*

9 Hymenium inspersed with oil droplets; epithecium bright green to bluish-green                                 *L. patavina*

- Hymenium lacks oil droplets; epithecium bluish-green, blackish-green to olivaceous or reddish-brown                        *L. stigmatea*

10 On bark or rotting wood                                                               11

- On rocks, occasionally on soil or wood                                                       14

11 Parathecium purple; apothecial margin black or shiny golden; thallus sometimes sorediate                            *L. mandshurica*

- Parathecium yellowish to reddish-brown; apothecial margin black; thallus esorediate                                   12

12 Hymenium inspersed with oil droplets; containing arthothelin, thiophanic acid, thuringione and granulosin                   *L. elaeochroma*

- Hymenium lacks oil droplets; arthothelin, thiophanic acid, thuringione and granulosin absent                               13

13 Lichexanthone present as a major compound, capistratone absent                                        *L. tumidula*

- Capistratone present as a major compound, lichexanthone absent                                        *L. euphorea*

14 Diploicin and thuringione present                                                    *L. carpathica*

- Diploicin absent                                                                 15

15 Capistratone present as a major compound, sometimes containing aotearone and isoarthothelin                         *L. effugiens*

- Capistratone absent                                                                16

16 Thiophanic acid present as a major compound; on rocks, soil, wood or bark                                    *L. asema*

- Arthothelin as a major compound; on rocks                                                 *L. elaeochromoides*

## Figures and Tables

**Figure 2:**
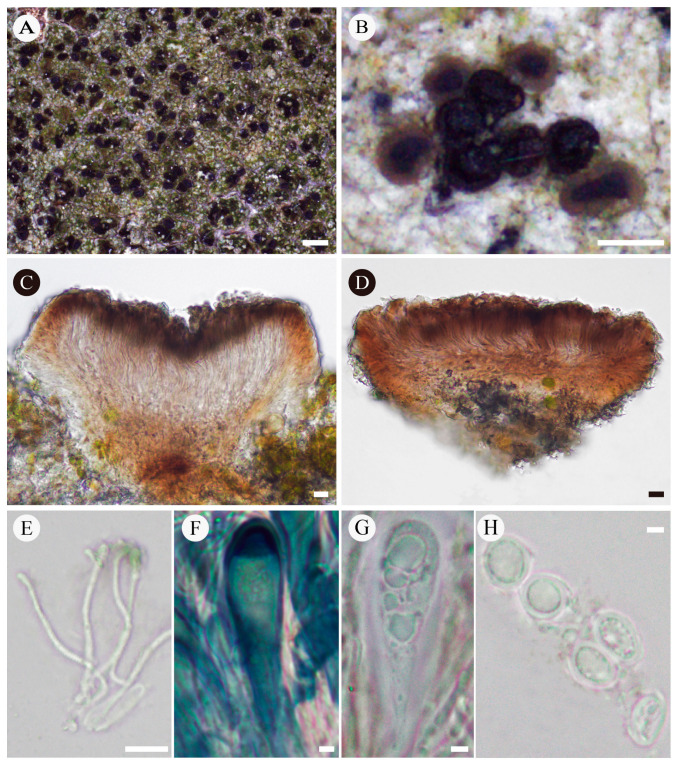
*Lecidella biformis* ((**A**,**C**,**E**,**F**) are from ANUB 2352; (**B**,**D**) are from ANUB 2406; (**G**,**H**) are from ANUB 2412). (**A**) Lichen thallus, habit. (**B**) Apothecia in two morphological types. (**C**) Vertical sections of apothecia (disc brown with paler margin). (**D**) Vertical sections of apothecia (disc and margin purely brown). (**E**) Paraphyses. (**F**) Ascus (in I reagent). (**G**) Ascus and ascospores. (**H**) Ascospores. Scale bars: 0.5 mm (**A**); 0.2 mm (**B**); 20 µm (**C**,**D**); 5 µm (**E**–**H**).

**Figure 3:**
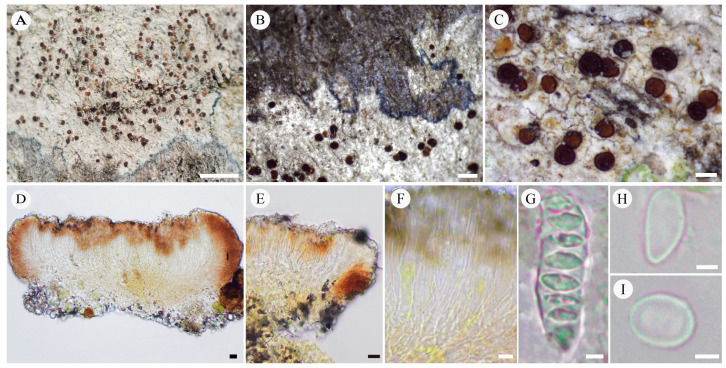
*Lecidella versicolor* (ANUB1890). (**A**) Lichen thallus, habit. (**B**). Prothallus. (**C**) Apothecia. (**D**) Vertical sections of apothecia (apothecia when mature). (**E**) Vertical sections of apothecia (apothecia when young). (**F**) Anastomosing paraphyses (in K reagent). (**G**) Eight-spored ascus. (**H**,**I**) Ascospores. Scale bars: 0. 5 mm (**A**,**B**); 0.2 mm (**C**); 20 µm (**D**–**F**); 5 µm (**G**–**I**).

## Data Availability

All of the data that support the findings of this study are available in the main text.
